# High-Level Antimicrobial Efficacy of Representative Mediterranean Natural Plant Extracts against Oral Microorganisms

**DOI:** 10.1155/2014/839019

**Published:** 2014-06-26

**Authors:** Lamprini Karygianni, Manuel Cecere, Alexios Leandros Skaltsounis, Aikaterini Argyropoulou, Elmar Hellwig, Nektarios Aligiannis, Annette Wittmer, Ali Al-Ahmad

**Affiliations:** ^1^Department of Operative Dentistry and Periodontology, Center for Dental Medicine, University of Freiburg, Hugstetter Straße 55, 79106 Freiburg, Germany; ^2^Department of Pharmacognosy and Natural Product Chemistry, Faculty of Pharmacy, National and Kapodistrian University of Athens, 15771 Athens, Greece; ^3^Department of Hygiene and Microbiology, Albert-Ludwigs University, 79104 Freiburg, Germany

## Abstract

Nature is an unexplored reservoir of novel phytopharmaceuticals. Since biofilm-related oral diseases often correlate with antibiotic resistance, plant-derived antimicrobial agents could enhance existing treatment options. Therefore, the rationale of the present report was to examine the antimicrobial impact of Mediterranean natural extracts on oral microorganisms. Five different extracts from *Olea europaea*, mastic gum, and *Inula viscosa* were tested against ten bacteria and one *Candida albicans* strain. The extraction protocols were conducted according to established experimental procedures. Two antimicrobial assays—the minimum inhibitory concentration (MIC) assay and the minimum bactericidal concentration (MBC) assay—were applied. The screened extracts were found to be active against each of the tested microorganisms. *O. europaea* presented MIC and MBC ranges of 0.07–10.00 mg mL^−1^ and 0.60–10.00 mg mL^−1^, respectively. The mean MBC values for mastic gum and *I. viscosa* were 0.07–10.00 mg mL^−1^ and 0.15–10.00 mg mL^−1^, respectively. Extracts were less effective against *C. albicans* and exerted bactericidal effects at a concentration range of 0.07–5.00 mg mL^−1^ on strict anaerobic bacteria (*Porphyromonas gingivalis, Prevotella intermedia, Fusobacterium nucleatum, and Parvimonas micra*). Ethyl acetate *I. viscosa* extract and total mastic extract showed considerable antimicrobial activity against oral microorganisms and could therefore be considered as alternative natural anti-infectious agents.

## 1. Introduction

The Hippocratic statement “Nature, without instruction or knowledge, does what is necessary” wisely acknowledges the healing contribution of the vegetable kingdom to the treatment of various diseases. The potential of a small fraction of natural plant extracts to cure serious infections of the human body has been well established to date [1–4]. In particular, various Mediterranean natural herb products have been screened profitably for their antioxidant and antimicrobial effects [5–8].* Origanum dictamnus*,* Olea europaea*, Chios mastic gum,* Inula viscosa*,* Petroselinum crispum*, and* Sideritis* spp. are some of the most well-known examples [7, 9, 10]. Nevertheless, the biological behavior of over 300.000 existing plant species needs to be further studied [11].

The remarkable immunostimulatory activity of several plant species is also responsible for the low occurrence of infectious processes in their wild counterparts. The most known broad-spectrum defense mechanisms of plant-originated antimicrobial agents are related to the presence of “phytoalexins.” The latter are small-molecule antibiotics (molecular weight: MW < 500) and according to their classification include polyphenols, flavonoids, terpenoids, and glycosteroids [12]. In order to conquer the pathogenic attackers, the aforementioned mild antimicrobials usually act synergistically. Moreover, the production of callose, a sugar polymer with (1–3)-*β*-D-glucan subunits, at the microbial invasion sites on the plant cell wall constitutes a typical pathogen-specific response of vegetable organisms [13]. The secretion of resistance (*R*) proteins triggered by avirulence (Avr) genes in the presence of attacking microorganisms is also another microbe-specific defense of plants [14].

Bacteria, viruses, and fungi are the cause of numerous infectious diseases. They are official residents of the human body and are capable of forming biofilms, dynamic microbial networks, on human substrata [15]. In the oral cavity in particular, more than 700 microbial species have been recovered, either being in a planktonic form or being embedded in a polysaccharide-affluent extracellular layer [16]. Despite the unfriendly conditions on tooth surfaces and gingival tissues, oral microorganisms are skilled at surviving through complicated physicochemical intercommunication patterns with the oral substrata [17]. The harmful microbial impact on these surfaces results in the genesis of caries, gingivitis, or periodontitis.

Recent years have seen a focus in oral microbiology research on the elucidation and elimination of biofilm-related dental diseases [18, 19]. Microbial biofilm communities have been found to be up to 1000 times more resistant to antibiotics against their equivalent planktonic cultures, while multidrug-resistant oral microorganisms have been identified [19]. The presence of vancomycin-resistant enterococci (VRE) and methicillin-resistant* Staphylococcus aureus* (MRSA) underlines the limits of antibiotics in facing this primary health hazard with regard to the oral cavity [20]. Furthermore, an endocarditis-associated* Enterococcus faecalis* virulence gene named efaA has been recovered from therapy-resistant strains in root canals [21].

The need to discover new efficient treatment strategies against oral microbial species has raised interest in natural vegetable sources. Novel antimicrobial agents of plant origin aim to reintroduce traditional treatment paradigms to modern medicine. Indeed, plants exhibit a remarkable pharmaceutical range due to their secondary metabolism-mediated chemical responses to various microorganisms and the synergistic paradigms they develop. The latter prevent microbes from resisting antibiotic monotherapy and, therefore, becoming untreatable.

The aim of the present report was to examine the antimicrobial activity of natural plant and fruit extracts of Mediterranean origin against various microbial species. More specifically, five different extracts from olive leaves, table olives, mastic gum, and* Inula viscosa* (Syn.* Dittrichia viscosa*) were screened against a panel of nine relevant pathogenic microorganisms, which constitute typical residents of the oral microflora, including one strain of* Candida albicans*. Additionally,* Staphylococcus aureus *and* Escherichia coli*, normally a part of skin and intestinal flora, served as reference bacterial strains. The null hypothesis of this report was that the aforementioned extracts have no antimicrobial impact on the tested microorganisms. For this purpose, two antimicrobial assays—the minimum inhibitory concentration (MIC) assay and the minimum bactericidal concentration (MBC) assay—were utilized.

## 2. Materials and Methods

### 2.1. Extraction Process

#### 2.1.1. Olive Leaves

*Olea europaea* leaves were dried in a well-ventilated shady place and subsequently stored in a dark room. Before their extraction, the leaves were ground using an Allen West type SCIS grinder with a sieve of 3 mm. The leaves (3.5 Kg) were extracted by mechanical stirring for 12 h with acetone (2 × 2.5 L). The extract was evaporated completely and washed with a mixture of CH_2_Cl_2_/MeOH 98 : 2 (3 L). The insoluble material was separated and dried under reduced pressure, producing a yellow powder (360 g) containing 60% oleuropein [22].

#### 2.1.2. Table Olive Processing Wastewater

Olive fruits were cured for a period of 4 months, using an aqueous solution of 8% NaCl (w/v). The water extract (400 mL) was applied to Amberlite XAD-4 resin and the column eluted with 96% ethanol. After desorption, ethanol was evaporated by drying at 40°C and the phenolic fraction (1.34 g) was recovered [22, 23].

#### 2.1.3. Conventional Extraction of Mastic Gum

A quantity of mastic gum (500 g) was diluted in ethyl acetate (500 mL). 1,500 mL of methanol was then added. After a period of 2 days, a layer of poly-*β*-myrcene (150 g) was decanted. Filtration was applied in order to obtain a clear supernatant solution and the solvent mixture was evaporated in a rotary evaporator at 45°C with an 80 kPa vacuum (extraction A). The resulting semisolid residue was dried in a desiccator at 70°C and 1,000-mbar vacuum and resulted in a white powder (350 g) [24].

#### 2.1.4. *Inula viscosa*

*Inula viscosa *(Asteraceae) was extracted with pressurized liquid extraction. For that purpose, a Dionex accelerated solvent extraction (ASE) 300 System (Dionex, Sunnyvale, CA) with 100 mL stainless steel vessels was used. Specifically, 20 g of ground *I*. viscosa aerial parts was placed into tubular extraction cells. These were then placed into the carousel and the samples were extracted under the specified conditions: temperature: 70°C, pressure: 120 bar (preset by the instrument), preheat time: 1 min, heat time: 5 min, 2 extraction cycles of 5 min static time each, flush volume: 100%, and purge: 120 sec. Separate preparations of ethyl acetate extracts and methanol extracts were made. Analytical grade ethyl acetate and methanol were used and were evaporated to dryness under reduced pressure using a rotary evaporator (Buchi Rotavapor R-200) at 40°C. The obtained yields were 2.08 gr for the ethyl acetate extraction and 3.51 gr for the methanol.

### 2.2. Chemical Analysis of Extracts

The qualitative and quantitative determination of the tested extracts (olive leaves, table olive processing wastewater, and* Inula viscosa*) was performed in a HPLC-DAD system: Thermo Finnigan HPLC system (Thermo Finnigan, San Jose, CA) coupled with a Spectral System UV6000LP PDA detector. A two-solvent gradient method was used: (A) H_2_O and (B) acetonitrile. The flow rate was set at 1 mL/min and the following elution program was applied: 0–60 min linear gradient from 0% A to 50% B; 60–65 min linear gradient to 100% B; 65–70 min 100% B isocratic; 70–75 min linear gradient to 0% B; 75–85 min 0% B isocratic. The analysis was performed at 25°C and the injection volume was 20 *μ*L. The detection was done at 280 nm and the column used was Supelco Analytical Discovery HS C18 (25 cm × 4.6 mm i.d., 5.0 *μ*m). Standard solutions of oleuropein and hydroxytyrosol were prepared and run under the same conditions in the case of olive leaves and table olive processing wastewater extracts [22, 23].

Concerning mastic gum the tested extract was divided into two fractions, an acidic and a neutral one. The acidic fraction after several chromatographic separations afforded the major triterpenic acids: oleanonic acid, moronic acid, 24Z-masticadienonic acid, 24Z-isomasticadienonic acid, 24Z-masticadienolic acid, and 24Z-isomasticadienolic acid. The neutral fraction afforded five neutral triterpenic compounds: tirucallol, dammaradienone, 28-norolean-12-en-3-one, oleanonic aldehyde, and oleanolic aldehyde. All the above constituents were identified by NMR (1H, 13C, COSY, HMQC, and NOESY) and MS and by comparison with data in the literature. Nuclear magnetic resonance (NMR) spectra were recorded on Bruker DRX 400 and Bruker AC 200 (50.3 MHz for 13C NMR) instruments at 295 K. Gas chromatography mass spectroscopy (MS) analysis was performed on a Finnigan GCQ Plus mass spectrometer [22].

### 2.3. Bacterial and Fungal Strains

A total of ten bacterial strains from eight different species and one* Candida albicans* strain were investigated. Eight of the tested bacterial strains and* C. albicans *occur in the oral flora, whereas* Staphylococcus aureus* and* Escherichia coli* are mainly recovered from the skin and intestinal flora, respectively. The latter two were used as reference microorganisms to specifically compare the oral inhibitory effect of natural extracts to their general antimicrobial activity. In particular,* Streptococcus mutans* DSM 20523,* Streptococcus sobrinus* DSM 20381,* Streptococcus oralis* ATCC 35037,* Enterococcus faecalis* ATCC 29212, and* S. aureus* ATCC 25923 are facultative anaerobic Gram-positive species, whereas* E. coli* ATCC 25922 is also facultative anaerobic but has a Gram-negative cell wall.* Porphyromonas gingivalis* W381,* Prevotella intermedia* ATCC 25611,* Fusobacterium nucleatum* ATCC 25586, and* Parvimonas micra* ATCC 23195 are obligate anaerobes. All bacterial and fungal strains were kindly supplied by the Division of Infectious Diseases and the Institute of Medical Microbiology and Hygiene of the Albert-Ludwigs University, Freiburg. The microorganisms were deposited at −80°C in basic growth medium containing 15% (v/v) glycerol until their use.

### 2.4. Determination of the Minimum Inhibitory Concentration (MIC)

First, an overnight culture of each bacterial and fungal strain was prepared according to the CLSI guidelines [25, 26]. Each dilution was plated on Columbia blood agar (CBA) plates or yeast-cysteine blood agar (HCB) plates. Facultative anaerobic bacteria and* C. albicans* were incubated on CBA agar plates at 37°C and 5%–10% CO_2_ atmosphere for 24 h. Anaerobic bacteria were placed on HCB agar plates at 37°C for 48 h (anaerobic chamber, Genbox BioMérieux SA, Marcy/Etoile, France). For the microdilution assay, all facultative anaerobic strains were inoculated in Mueller-Hinton broth (MHB), anaerobic bacteria in Wilkins-Chalgren broth (WCB), and* C. albicans *in Sabouraud dextrose broth (SDB), each to be tested at 10^6^ colony forming units (CFU) mL^−1^. Afterwards, appropriate volumes of the MHB/WCB/SDB microbial cultures were transferred into a 96-well microtiter-plate using a multichannel pipette. The prepared natural extracts were then dissolved in dimethyl sulfoxide (DMSO, Sigma, Steinheim, Germany) and diluted in aqua destillata. All extract solutions in DMSO were screened in a concentration series ranging from 10 mg mL^−1^ to 0.02 mg mL^−1^ at dilution levels starting from 2-fold to 512-fold. The experiments were performed in duplicate. A 0.5/1 A McFarland standard suspension was prepared in normal saline for bacteria and fungi, respectively. Each well of the 96-well microtiter-plate had a total volume of 200 *μ*L. In order to exclude potential antimicrobial effects of the DMSO residuals, a dilution series of DMSO was examined in parallel. Wells containing solely MHB/WCB/SDB or 0.2% chlorhexidine (CHX) served as positive and negative controls for bacterial growth, respectively. The possibility of contamination was minimized by using sterile MHB/WCB/SDB. Thereafter, facultative anaerobic bacteria and* C. albicans* were incubated at 37°C and 5%–10% CO_2_ atmosphere for 24 h, while anaerobic bacteria were kept at 37°C for 48 h (anaerobic chamber, Genbox BioMérieux SA, Marcy/Etoile, France). All assays for each bacterial and fungal strain were performed at least in duplicate. The highest minimum inhibitory concentration (MIC) values were taken into consideration in case the MIC values of a specific strain were not identical. MIC was defined as the lowest concentration of each natural extract at which visible inhibition of bacterial growth was induced and was thus expressed by the percentage of bacterial growth at that particular concentration. The inhibitory impact of DMSO was taken into consideration if bacterial growth was observed in the cotested DMSO dilution series.

### 2.5. Determination of the Minimum Bactericidal Concentration (MBC)

The minimum bactericidal concentration (MBC) was also assessed according to the CLSI guidelines [25, 26]. After completion of the MIC assay, the aforementioned 96-well microtiter-plates were further incubated for MBC testing. In brief, 10 *μ*L from each well containing the tested natural extract concentration series was plated on Columbia blood agar (CBA) or yeast-cysteine blood agar (HCB) plates. In particular, the facultative anaerobic bacteria and* C. albicans* were incubated on CBA plates at 37°C and 5%–10% CO_2_ atmosphere for 2 days. Strictly anaerobic bacteria were cultivated on HCB plates at 37°C for 5 days (anaerobic chamber, Genbox BioMérieux SA, Marcy/Etoile, France). The colony forming units (CFU) were determined visually. The MBC was defined as the concentration at which a three-log decrease in bacterial growth (= 99.9%) was detected compared to the positive control.

## 3. Results

### 3.1. *Olea europaea*

Two extracts produced from olive processing byproducts were tested. Extraction of olive leaves with acetone afforded a polar fraction which was defatted with a mixture of CH_2_Cl_2_/MeOH 98 : 2. The obtained extract contained 60% oleuropein. The extract coming from table olive processing wastewater contained as its main compound, the degradation product of oleuropein, hydroxytyrosol, in a percentage around 15%.

The mean MIC and MBC values for each of the* O. europaea* extracts as well as the tested bacterial/fungal strains are presented in Table 1.

In general, table olive extract was more active than olive leaf extract. It was effective against almost all of the tested microorganisms, with a mean concentration range of 0.15 mg mL^−1^ (*Porphyromonas gingivalis, Parvimonas micra*) to 10.00 mg mL^−1^ (*Candida albicans*). For the facultative anaerobic bacteria MIC values varied between 1.25 mg mL^−1^ (*Streptococcus mutans*,* Streptococcus oralis*) and 5.00 mg mL^−1^ (*Enterococcus faecalis*). The MBC values of the table olive extract ranged from 0.60 to 10.00 mg mL^−1^. Obligate anaerobes (*Prevotella intermedia*,* P. micra*) were more easily eradicated (0.60 mg mL^−1^) when compared to* E. faecalis* and* C. albicans* (10.00 mg mL^−1^).

Olive leaf extracts showed a milder inhibitory effect against oral pathogens. The MIC values of the eradicated microbial strains were between 0.07 mg mL^−1^ (*S. oralis*) and 10.00 mg mL^−1^ (*C. albicans*,* Escherichia coli*). The MBC values demonstrated the persistence of facultative anaerobes in the presence of* O. europaea* leaf extracts (10.00 mg mL^−1^). However,* P. gingivalis* was inhibited by 0.60 mg mL^−1^ of the extract, while 1.25 mg mL^−1^ killed 99.9% of* Fusobacterium nucleatum *and* P. micra*.

### 3.2. Mastic Gum

Table 2 summarizes the MIC and MBC values of the tested mastic gum extract for all screened microbial strains. A total mastic extract without polymer was prepared after removal of the contained insoluble polymer. The extensive characterization of the extract by NMR and MS revealed that it contained triterpenic acids, triterpenic alcohols, and aldehydes.

Total mastic extract was effective against all of the microorganisms with MIC values ranging from 0.02 mg mL^−1^ (*P. gingivalis*) to 10 mg mL^−1^. The mean MBC values were between 0.07 mg mL^−1^ (*P. gingivalis*,* P. micra*) and 10.00 mg mL^−1^ (*S. mutans*,* S. sobrinus*,* E. faecalis*,* C. albicans*, and* E. coli*). Extract concentrations between 0.07 and 2.50 mg mL^−1^ exerted bactericidal effect mainly on strict anaerobic, Gram-negative bacteria (*P. gingivalis*,* P. intermedia*, and* F. nucleatum*).

### 3.3. *Inula viscosa*

Table 3 summarizes the range of MIC and MBC values of the two tested* I. viscosa *extracts against the selected oral bacterial/fungal strains. Aerial parts of* I. viscosa* were extracted with two solvents of different polarity, following a separate extraction. Ethyl acetate extract afforded medium polarity compounds, mainly flavonoids such as 3-*O*-acetylpadmatin, 7-*O*-methylaromadendrin, hispidulin, apigenin, luteolin, sesquiterpenic compounds, and triterpenoids. While the methanol extract contained some common compounds with the ethyl acetate extract, phenolic acids and flavonoids were also detected.

Among the two* I. viscosa* extracts, ethyl acetate extract presented a more robust antimicrobial effect against the screened microorganisms compared to the methanol extract. Under its influence, a mean inhibitory concentration range of 0.07 mg mL^−1^ (*P. gingivalis*) to 2.50 mg mL^−1^ (*S. sobrinus*,* E. faecalis*, and* E. coli*) was observed. The MBC values of the ethyl acetate extract varied from 0.15 to 5.00 mg mL^−1^. Obligate anaerobes such as* P. gingivalis *(0.15 mg mL^−1^) were efficiently eliminated by reduced extract concentrations as compared to the more persistent* E. faecalis* and* E. coli *strains (5.00 mg mL^−1^).

The* I. viscosa* methanol extract also had a considerable inhibitory impact on oral bacteria and fungi. The MIC values of the eradicated microorganisms ranged between 0.15 mg mL^−1^ (*P. gingivalis*) and 10.00 mg mL^−1^ (*E. coli*). The MBC values ranged from 0.30 to 10.00 mg mL^−1^. Hence,* P. gingivalis *was eliminated by 0.30 mg mL^−1^ of the extract, while 0.60 mg mL^−1^ induced a three-log reduction in bacterial growth for* P. intermedia *and* S. oralis*.

## 4. Discussion

Taking the antibiotic resistance of oral biofilms into consideration, the present study aimed to introduce novel plant-derived antimicrobial agents in the treatment of therapy-persistent dental diseases. Hence, the antimicrobial impact of five different Mediterranean natural plant and fruit extracts was investigated on representative oral bacterial strains and* C. albicans*. To the best of our knowledge, this is the first report on the antimicrobial efficacy of* Olea europaea*, mastic gum, and* Inula viscosa* against microorganisms which belong to the commensal flora of the oral cavity.

In this study, the tested* O. europaea* extracts were found to be especially effective against Gram-negative, anaerobic periodontal pathogens such as* Porphyromonas gingivalis*,* Prevotella intermedia*, and* Fusobacterium nucleatum. *Indeed, there are several reports on the excellent antibacterial activity of* O. europaea* extracts and pure compounds using diverse microbial species [7, 27–29]. Sudjana et al. substantiated the narrow-spectrum antibacterial action of commercial olive leaf extract against* Campylobacter jejuni*,* Helicobacter pylori*, and* Staphylococcus aureus,* as well as against methicillin-resistant* S. aureus* (MRSA) [30]. This highlights the regulatory interference of* O. europaea* extracts with gastric Gram-negative microorganisms such as* C. jejuni* and* H. pylori*. In another report olive leaf extracts succeeded in eliminating the Gram-negative* Escherichia coli*,* Pseudomonas aeruginosa*, and* Klebsiella pneumoniae* [31]. However, Gram-negative microorganisms are often resistant to conventional antimicrobial drugs because of their expression of active efflux pumps [32]. Their tenacious nature is also assisted by the release of degrading enzymes and molecular metamorphosis of antibiotic targets [33]. Moreover, the overdelicate structure of Gram-negative bacteria, which involves structural discrepancies between the cell wall and exterior membrane, influences their susceptibility to various antimicrobial agents [34].

Furthermore, table olive extract presented milder antistaphylococcal activity, suggesting an additional impact on Gram-positive bacteria. A previous report described the antimicrobial efficacy of* O. europaea* leaves towards the Gram-positive* Bacillus cereus *[27]. The exact infiltration and destruction mechanism of the Gram-positive cell wall by* O. europaea *extracts probably implies the presence of antiquorum sensing (QS) compounds [35]. Since the pathogenicity of Gram-positive microorganisms such as* S. aureus* is based on small peptides named “quormones” triggered by the* agr *operon or analogous QS communication patterns,* O. europaea* could assumingly interfere with their action [36]. Contrary to the results of an earlier study,* Candida albicans* was not found susceptible to table olive extract [27]. The conflicting outcomes can be attributed to the differing extraction methods which were used, including boiling. However, which of the numerous phenolic or other compounds was responsible for this favorable effect remains unknown. In the present study, the two main compounds of the extracts were oleuropein in olive leaves and hydroxytyrosol in table olive processing wastewater. A lot of studies claim the strong antimicrobial activity of the two compounds [22, 37].

Mastic gum constitutes a stem-derived resinous exudate of the Mediterranean tree* Pistacia lentiscus *var.* chia. P. gingivalis*,* P. intermedia*,* F. nucleatum*, and* P. micra *demonstrated high susceptibility toall of the mastic gum extracts studied. The numerous reports on the favorable antimicrobial properties of mastic gum also underline its effectiveness against various pathogens [6, 24, 38–40]. More specifically, a commercial product containing mastic in the liquid form (2%) showed a narrow antibacterial spectrum against Gram-negative* P. gingivalis* and* Prevotella melaninogenica *[40]. Gram-positive bacteria (*Streptococcus mutans*,* S. aureus*) as well as fungi (*C. albicans*) were not influenced. Takahashi et al. confirmed a decrease in the total number of bacterial salivary colonies after 4 hours of chewing mastic gum [41]. Since side effects such as tooth discoloration and enhanced cell toxicity accompany the application of conventional oral antibacterial chemicals such as chlorhexidine (CHX), the introduction of mastic-derived mouth rinses could promote antiplaque activity with minimal drawbacks.

In general, mastic gum is composed of various volatile ingredients, the most important of which are *α*-pinene, *β*-myrcene, *β*-caryophyllene, *β*-pinene, and limonene [6]. The herein tested extract, though, contained mostly triterpenic acids, which could be the active principles. It was previously found that total mastic extract without polymer containing mostly major triterpenic acids can efficiently eliminate other persistent microorganisms such as* H. pylori*. Reduced activity was observed for the triterpenic alcohols and aldehydes [24]. The additionally contained triterpenic alcohols and aldehydes have not showed a respective antimicrobial action. Koutsoudaki et al. detected moderate to high susceptibility of* B. subtilis* and* S. aureus* to *β*-myrcene, while* E. coli *exhibited no sensitivity to the substance [39]. Furthermore, *β*-pinene proved to be ineffective against* E. coli* and* S. aureus, *whereas* B. subtilis* showed only a mild response. These varying outcomes suggest that a synergistic activity of the different components of mastic gum extracts may be responsible for its antimicrobial action. Thus, the results of the present study indicate that the interdependent antimicrobial activity of the particular mastic ingredients focuses on eradicating therapy-resistant Gram-negative oral microorganisms.

In this study,* I. viscosa* was highly effective against not only Gram-negative anaerobic pathogens, but also streptococci (*S. mutans*,* S. oralis*). Although its advantageous effects in the oral cavity were demonstrated here for the first time, there are many reports appraising its general antibacterial, antifungal, and antiviral properties [42–45]. The difference between the activities of the two extracts could be attributed to the different chemical profile. Generally the two extracts contain a lot of common metabolites; however, the most polar ones are found at the methanol extract. In a previous report, methanol extracts of the plant demonstrated effective antimicrobial behavior against Gram-positive bacteria such as* S. aureus,* and* E. faecalis*,* C. albicans*, and* Candida tropicalis* were also found to be sensitive to* I. viscosa*, whereas Gram-negative bacteria were less susceptible [45]. The antimicrobial impact of* I. viscosa* was also observed on* Bacillus cereus* and* Salmonella typhimurium*. In addition, it was revealed that a compound of the plant named 3,3′-di-O-methylquercetin can deteriorate the cytoplasmic membranes after penetrating various bacterial cell walls [44]. Moreover, Wang et al. reported that* I. viscosa* presented fungicidal properties against pathogens of the families Oomycetes, Ascomycetes, and Basidiomycetes [46]. This is in agreement with the findings of this study, in which* I. viscosa *exerted bactericidal effects at a concentration range of 2.50–5.00 mg mL^−1^ towards* C. albicans*.

Medicinal plants have proven to be a bountiful reservoir of numerous biologically active components with advanced antibacterial properties. It is well known that synthetic chemicals, which are commonly used in dental products such as toothpastes and oral mouthwashes, can induce tooth discoloration, cell toxicity, regurgitation, or diarrhea [47, 48]. Thus, plant extracts are considered to be potent alternative compounds for the new generation of oral pharmaceuticals. In fact, novel plant-derived constituents of dental chemotherapeutics (mouth rinses, irrigation solutions, and intracanal medicaments) are crucial future sources of the dental industry specializing in plaque-control strategies, since they allow for efficient treatment regarding antibiotic-resistant pathogens, immunocompromised individuals, and financially weak developing countries.

## 5. Conclusion

In conclusion, the results of this report promote interest in the discovery of alternative natural compounds with antimicrobial activity against therapy-resistant oral microorganisms. Overall, extracts from* O. europaea*, mastic gum, and* I. viscosa* were active against the tested oral pathogens, especially Gram-negative anaerobic bacteria, and could therefore be considered as alternative natural anti-infectious agents which could be used against periodontitis.

## Figures and Tables

**Table 1:** Antimicrobial activity in mg mL^−1^ of olive leaf and table olives extracts (*O. europaea*).

*Ol* *ea* *europaea*						
Sample	Olive leaf extract		Table olives extract		DMSO (%)	
c/mg mL^−1^	MIC∗	MBC∗∗	MIC	MBC	MIC	MBC
*Streptococcus mutans* DSM 20523	1.25	NA	1.25	5.00	5.00	40.00
*Streptococcus sobrinus* DSM 20381	2.50	NA	2.50	5.00	20.00	20.00
*Streptococcus oralis* ATCC 35037	0.07	NA	1.25	2.50	10.00	20.00
*Enterococcus faecalis* ATCC 29212	0.60	NA	5.00	NA	20.00	80.00
*Candida albicans* DSM 1386	NA	NA	NA	NA	10.00	10.00
*Escherichia coli* ATCC 25922	NA	NA	5.00	NA	20.00	40.00
*Staphylococcus aureus* ATCC 25923	2.50	5.00	2.50	2.50	20.00	40.00
*Porphyromonas gingivalis* W381	0.30	0.60	0.15	1.25	5.00	20.00
*Prevotella intermedia* ATCC 25611	2.50	5.00	0.30	0.60	2.50	5.00
*Fusobacterium nucleatum* ATCC 25586	0.60	1.25	0.60	2.50	10.00	10.00
*Parvimonas micra* ATCC 23195	0.30	1.25	0.15	0.60	10.00	20.00

NA: no activity observed; MIC or MBC was measured at 10.00 mg mL^−1^.

*MIC: extract concentration at which the optical density (OD) measurement revealed minimal bacterial growth.

**MBC: extract concentration at which a three-log reduction (99.9%) of the bacterial growth was induced.

**Table 2:** Antimicrobial activity in mg mL^−1^ of four mastic gum extracts.

Mastic gum				
Sample	Total mastic extract		DMSO (%)	
c/mg mL^−1^	MIC∗	MBC∗∗	MIC	MBC
*Streptococcus mutans* DSM 20523	NA	NA	5.00	40.00
*Streptococcus sobrinus* DSM 20381	NA	NA	10.00	20.00
*Streptococcus oralis* ATCC 35037	2.50	5.00	10.00	20.00
*Enterococcus faecalis* ATCC 29212	NA	NA	10.00	80.00
*Candida albicans *DSM 1386	NA	NA	10.00	10.00
*Escherichia coli* ATCC 25922	NA	NA	10.00	10.00
*Staphylococcus aureus* ATCC 25923	2.50	5.00	20.00	80.00
*Porphyromonas gingivalis* W381	0.02	0.07	10.00	20.00
*Prevotella intermedia* ATCC 25611	0.07	0.30	2.50	5.00
*Fusobacterium nucleatum* ATCC 25586	2.50	2.50	10.00	20.00
*Parvimonas micra* ATCC 23195	0.60	0.60	2.50	10.00

NA: no activity observed; MIC or MBC was measured at 10.00 mg mL^−1^.

*MIC: extract concentration at which a three-log reduction (99.9%) of the bacterial growth was induced.

**MBC: extract concentration at which a three-log reduction (99.99%) of the bacterial growth was induced.

**Table 3:** Antimicrobial activity in mg mL^−1^ of *I*. *viscosa* extracts.

*In* *ul* *a* *viscosa*						
Sample	Ethyl acetate extract		Methanol extract		DMSO (%)	
c/mg mL^−1^	MIC∗	MBC∗∗	MIC	MBC	MIC	MBC
*Streptococcus mutans* DSM 20523	0.15	1.25	1.25	5.00	5.00	40.00
*Streptococcus sobrinus* DSM 20381	2.50	2.50	2.50	5.00	20.00	20.00
*Streptococcus oralis* ATCC 35037	0.30	0.30	0.60	0.60	10.00	20.00
*Enterococcus faecalis* ATCC 29212	2.50	5.00	5.00	5.00	20.00	80.00
*Candida albicans* DSM 1386	1.25	2.50	5.00	5.00	10.00	10.00
*Escherichia coli* ATCC 25922	2.50	5.00	NA	NA	20.00	40.00
*Staphylococcus aureus* ATCC 25923	0.60	1.25	1.25	2.50	20.00	40.00
*Porphyromonas gingivalis* W381	0.07	0.15	0.15	0.30	5.00	20.00
*Prevotella intermedia* ATCC 25611	0.15	0.30	0.30	0.60	2.50	5.00
*Fusobacterium nucleatum* ATCC 25586	0.30	0.60	0.60	1.25	10.00	10.00
*Parvimonas micra* ATCC 23195	0.15	0.60	0.60	1.25	10.00	20.00

NA: no activity observed; MIC or MBC was measured at 10.00 mg mL^−1^.

*MIC: extract concentration at which the optical density (OD) measurement revealed minimal bacterial growth.

**MBC: extract concentration at which a three-log reduction (99.9%) of the bacterial growth was induced.
